# Effects of exercise training on neurological recovery, TGF-β1, HIF-1α, and Nogo-NgR signaling pathways after spinal cord injury in rats

**DOI:** 10.1016/j.clinsp.2023.100236

**Published:** 2023-07-27

**Authors:** Xubin Ji, Zhaowan Xu, Dayong Liu, Yangwang Chen

**Affiliations:** Department of Spinal Surgery, Weifang People's Hospital, Weifang, Shandong, PR China

**Keywords:** Spinal cord injury, Motor training, Neurological function, TGF-β1, HIF-1α

## Abstract

**Objective:**

To evaluate the effects of exercise training on neurological recovery, Growth Transforming Factor-β1 (TGF-β1), Hypoxia Inducible Factor-1α (HIF-1α), and Nogo-NgR signaling pathways after spinal cord injury in rats.

**Methods:**

Forty-eight male Sprague-Dawley rats were randomly divided into four groups: normal group, sham-operated group, model group, and training group. The rat spinal cord injury model was established using Allen's method, and the training group received exercise training on the 8th day postoperatively. The Basso, Beattie and Bresnahan (BBB) score, modified Tarlow score, and inclined plane test scores were compared in each group before injury and 1, 7, 14, 21 and 28 days after injury.

**Results:**

The BBB score and modified Tarlow score of the model group and the training group were 0 at the first day after the injury, and gradually increased on the seventh day onwards (*p* < 0.05). The BBB score and modified Tarlow score of the training group were higher than those of the model group at the 14th, 21st and 28th day (*p* < 0.05). The angles of the inclined plate at multiple time points after injury were lower in the model group and the training group than in the normal group and the sham-operated group (*p* < 0.05); The angles of the inclined plate at the 14th, 21st and 28th day after injury were higher in the training group than in the model group (*p* < 0.05).

**Conclusion:**

The mechanism of exercise training may be connected to the inhibition of the Nogo-NgR signaling pathway to promote neuronal growth.

## Highlights

• Exercise training can enhance motor function and improve the spinal cord.
• Exercise training can improve gastrocnemius muscle morphology.
• Exercise training can promote neurological recovery after spinal cord injury in rats.

## Introduction

A spinal cord injury, also known as an SCI, is a form of Central Nervous System (CNS) trauma that is typically brought on by mechanical trauma to the spinal cord or cauda equina. SCIs are known to have numerous problems below the level of injury, including motor, sensory, and autonomic dysfunction.^1^ As the disease worsens, SCI interferes with the dynamic interplay between peripheral sensory input and the spinal cord neuronal network, potentially leading to long-term functional problems.^2^ The repair of spinal cord injury has thus become a global emphasis in the domains of rehabilitation medicine, neuroscience, and orthopedic research. How to efficiently treat early SCI is of tremendous practical value.

Currently, clinical repair strategies regarding spinal cord injury are based on enhancing neurological plasticity, repairing myelin sheaths, and promoting axonal regeneration, among which increasing spinal cord plasticity is the basis for restoring neurological function.^3^ Exercise training is a common form of rehabilitation, and research on both humans and animals has shown that it can reduce secondary injury by inhibiting nerve cell apoptosis, promoting the secretion and release of factors related to nerve growth, and introducing bodily sensory signals to neurons in the spinal motor nerve center.^4^,^5^ Additionally, exercise training can improve the microenvironment of spinal cord tissues, stimulate neuronal pathways in the spinal cord, increase spinal cord plasticity, and facilitate neurological and motor function recovery to some extent, but the precise mechanism of action is still unclear.^6^ Axonal regeneration and derivation can be inhibited by localized oxidative stress, ischemia, and hypoxia brought on by SCI, which in turn impacts the neurological recovery process. Growth Transforming Factor-β1 (TGF-β1) is a component of bone healing and represents the state of bone repair following injury. Hypoxia Inducible Factor-1α (HIF-1α) is used to reflect the oxidative stress response and plays a regulatory role in biological behaviors such as apoptosis and angiogenesis.^7^ The Nogo-NgR signaling pathway can repair nerve injury function by regulating axon regeneration.^8^ To offer a foundation for clinical SCI treatment, this study created a rat spinal cord injury model using Allen's approach and examined the effects of exercise training on neurological recovery, TGF-β1, HIF-1α, and Nogo-NgR signaling pathways.

## Materials and methods

### Experimental animals and grouping

Forty-eight male healthy Sprague-Dawley (SD) rats (Beijing Viton Lever Laboratory Animal Technology Co., Ltd., SCXK (Beijing) 2020–0001), 8 weeks old, body mass 280±30 g, were randomly divided into 4 groups of normal groups, sham-operated group, model group and training group after 1 week of adaptive feeding, 12 rats in each group. The whole experimental procedure was in accordance with the relevant requirements in the Regulations of the People's Republic of China on the Administration of Laboratory Animals. The experiments involving animals in this study strictly followed the ARRIVE guidelines.^9^

## Study methods

### Animal model preparation

The rat spinal cord injury model was established by Allen's method: intraperitoneal anesthesia with 10% chloral hydrate (300 mg/kg) (Shanghai Lianshuo Biotechnology Co., Ltd., China). Under the supine position, a longitudinal incision was made with T10 as the center, exposing the T9-T11 spine. With a stainless-steel rod hitting T10, the height of the blow was 25 mm, the mass was 10 g, and the diameter of the injury was 3 mm. The modeling was considered successful if the rats showed spasmodic tail wagging, spasmodic shaking of both lower limbs and spinal cord congestion. In the sham-operated group, only the spinous process and the vertebral plate were removed. After successful modeling, the incision was sutured and 1 ml of penicillin (Beijing Solabao Technology Co., Ltd., China) was injected intramuscularly to prevent infection, and excretion was promoted by manual bladder squeezing. None of the above experimental operations caused fatal damage to the rats, and the experimental subjects could complete the experiment without any effect on the experimental results. Every procedure was approved by the Animal Care and Use Committee of the Weifang People's Hospital (protocol number NCT0256347).

### Exercise training

**Fig. 1:**
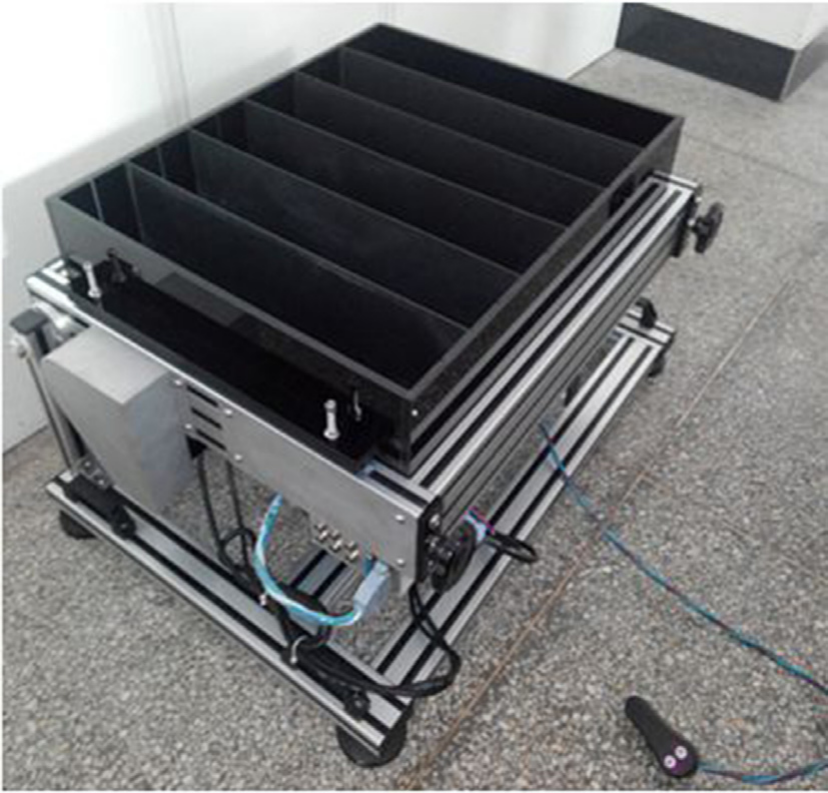
Picture of experimental setup designed for the exercise training protocols in rats.

The training group started weight-bearing walking training on the 8th postoperative day: the device was a rectangular body with two pieces of long glass to divide the runway into 3 runways evenly, and 4 iron bars were fixed transversely to the upper end of the device (Fig. 1). A 40-cm-long, 1-cm-wide harness was fixed on the bar, and a clip was attached to the other end of the harness to adjust the length of the harness to control the weight that the rats bear. The lumbosacral and the back of the rat were respectively clamped by the clamps, and the rats were placed on the exercise board for passive continuous walking. The weight loss device was custom-made. The weight was reduced each time equal to 20%‒40% of the rat's body weight. The speed of the treadmill was 8 m/min, and the training frequency was 1 time/d, 10 min/time, 6d/week.^10^

### Motor function assessment

1) Basso, Beattie and Bresnahan (BBB) score. The BBB scale is a 22-point scale, which was judged according to the neuromotor function of the hind limbs of the rats. The rats were free to move around for 5 min and observed the trunk movement, tail movement and gait coordination, etc. The scale ranged from 0‒21, with 0 for hind limb paralysis and no motor function, and 21 for complete normal function and movement, with a positive correlation between motor function and score, with higher scores representing better recovery of nerve function after spinal cord injury. 2) Modified Tarlow score. The evaluation items include hindlimb walking, weight-bearing walking, etc. No active activity of hind limb was scored as 0, no weight-bearing of the hind limb and small range of activity was scored as 1, no weight-bearing and walking of the hind limb, the frequent or strong activity of the hind limb was scored as 2, no correct gait but hind limb could support weight and walk 1‒2 steps forward was scored as 3, only mild impairment, weight-bearing and walking of the hind limb was scored as 4, and completely normal behavior was scored as 5. 3) Angles of the inclined plate. The rat was placed on a smooth wooden board with the body axis perpendicular to the longitudinal axis of the inclined board, and the board was gently raised by 5° each time, and the maximum angle at which the rat stayed on the inclined board for 5s was recorded. The above assessments were performed before injury, the 1st d, 7th d, 14th d, 21st d, and 28th d after injury.

### Light microscopic sampling and section preparation

All groups were sampled on the 28th day after the injury, perfused with 4% paraformaldehyde phosphate buffered solution in the heart, and the distal L5 spinal cord and either side of the gastrocnemius muscle were sampled. After dehydration with graded series alcohol, the sections were sliced longitudinally to 20 μm thickness and stained with HE staining. Morphological changes were observed under EM Cryo CLEM-type light microscopy (Leica Microsystems [Shanghai] Trading Co., Ltd., China), and 10 fields were randomly selected and observed under a high-power fluorescence microscope (200×).

### Electron microscopic sampling and image analysis

After perfusion with 4% paraformaldehyde phosphate buffer and fixation for 1 h, the spinal cord of segment L5 was taken and fixed using 4% glutaraldehyde phosphate buffer for 24 h. After fixation with 1% osmium tetroxide for 10 min, the specimens were processed. The semi-thin sections and ultra-thin sections were observed under a Hitachi H-7500 type transmission electron microscope (Hitachi Scientific Instruments (Beijing) Ltd., Japan) Images were analyzed using the Image-ProPlus 6.0 image analysis system (Media Cybernetics, Inc., USA) to obtain the cross-sectional area and diameter of muscle fibers.

### RT-qPCR

Rat spinal cord tissues were taken out, ground, and lysed under the protection of liquid nitrogen, and total RNA was extracted from the tissues by the Trizol method (Shenyang Wan Class Biotechnology Co., Ltd., China), and the RNA samples were reverse transcribed into cDNA by the reverse transcription kit (Wuhan Yipu Biotechnology Co., Ltd., China). RT-qPCR was performed using SYBR Prellix Ex TaqTM real-time PCR kit (TaKaRa, Japan), with GAPDH as the internal reference. The conditions were as follows: 95 °C for 5 min, 95 °C for 10 s, 60 °C for 30 s, 40 cycles, and 75 °C for 10 min at 4 °C. The mRNA expression of TGF-β1, HIF-1α, Nogo-A, NgR, and LINGO-1 was calculated by the 2^−△△CT^ method.

### Immunofluorescence staining method

Six randomly selected spinal cord sections from each rat were baked, dewaxed, hydrated, endogenous peroxidase removed, repaired, closed, and titrated with primary antibody (Microtubule Associated Proteins 1B [MAP1B] 1:200 dilution, Neuron Specific Enolase [NSE] 1:100 dilution, Vimentin [VIM] 1:300 dilution), protected from light with secondary antibody (FITC 1:200 dilution), restrained, and sealed in PBS saline. Sections were observed and photographed under a CKX41 inverted fluorescence microscope (Olympus, Japan).

### Western blot

On the 14th day after the injury, the spinal cord tissues of 5 rats in each group were randomly taken from the injured site, cut with tissue scissors, placed on ice in a frozen tube, and 1 μL PMSF lysate was added. Proteins were collected after cell lysis, quantified by the BCA method (Shanghai JiZi Biochemical Technology Co., Ltd., China), electrophoresed on SDS-PAGF gel, with 5% stacking gel at 40 V for 1 h, 10% separating gel at 60 V for 3.5 h, wet transferred at 14 V for 14 h with constant pressure, transferred on a PVDF membrane (Millipore, USA), and closed at room temperature for 2 h in 5% fat-free milk. Primary antibody (horseradish peroxidase-labeled sheep anti-rabbit IgG with sheep anti-mouse IgG, Abcam, UK) was added and shaken at room temperature for 2 h and incubated overnight at 4 °C. A secondary antibody (1:2000 dilution) was added and incubated for 2 h at room temperature for chemiluminescence detection using ECL reagents (Solebro Technology Co., Ltd., Beijing, China). GAPDH (1:1000 dilution) (ZhongShan JinQiao Biotechnology, Beijing, China) was used as the internal reference, and the Chemi Image 5500 automated electrophoresis gel imaging analyzer (Alpha Innotech, USA) was used to collect the results and calculate the grayscale values.

### Statistical analysis

SPSS 23.0 statistical analysis software was used to determine whether the data were normally distributed using Shapiroe-Wilk, and the measurement data conforming to normal distribution were expressed as ¯χ±*S*. One-way ANOVA was used for comparison between multiple groups, and further two-by-two comparisons were made using the LSD-*t*-test, with *p* < 0.05 being considered a statistically significant difference.

## Results

### BBB score

The BBB scores of the normal group and the sham-operated group at different time points were 21; the BBB scores of the model group and the training group were 0 at the 1st d after injury and gradually increased from 7th d onwards (*p* < 0.05), and the BBB scores of the training group at the 14th d, 21st d and 28th d were higher than those of the model group (*p* < 0.05) (Table 1).

**Table 1:** Comparison of BBB scores (¯χ±*S*, points).

Group	Pre-injury	Post-injury				
		1st d	7th d	14th d	21st d	28th d
Normal group	21.00±0.00	21.00±0.00	21.00±0.00	21.00±0.00	21.00±0.00	21.00±0.00
Sham-operated group	21.00±0.00	21.00±0.00	21.00±0.00	21.00±0.00	21.00±0.00	21.00±0.00
Model group	21.00±0.00	0.00±0.00^b^	3.86±0.84^b^	5.86±1.15^b^	10.53±3.32^b^	13.36±4.11^b^
Training group	21.00±0.00	0.00±0.00^b^	3.69±0.62^b^	10.63±2.14^a^^,^^b^	14.62±4.84^a^^,^^b^	17.32±3.32^a^^,^^b^

Note: Compared with the normal and sham-operated groups.

a *p* < 0.001; compared with model group.

b *p* < 0.001.

### Modified tarlow scores

Compared with the modified Tarlow scores at different time points in the normal and sham-operated groups, the differences were not statistically significant (*p* > 0.05); the modified Tarlow scores in the model group and the training group were all 0 at the 1st d after injury and showed a gradual increase from the 7th d onwards (*p* < 0.05), and the modified Tarlow scores at the 14th, 21st, and 28th d in the training group were all higher than those in model group (*p* < 0.05) (Table 2).

**Table 2:** Comparison of modified Tarlow scores (¯χ±*S*, points).

Group	Pre-injury	Post-injury				
		1st d	7th d	14th d	21st d	28th d
Normal group	5.00±0.00	5.00±0.00	5.00±0.00	5.00±0.00	5.00±0.00	5.00±0.00
Sham-operated group	5.00±0.00	4.89±0.08	4.98±0.02	5.00±0.00	5.00±0.00	5.00±0.00
Model group	5.00±0.00	0.00±0.00^a^	1.05±0.21^a^	2.21±0.42^a^	2.54±0.41^a^	2.96±0.42^a^
Training group	5.00±0.00	0.00±0.00^a^	1.14±0.19^a^	2.96±0.37^a^^,^^b^	3.34±0.52^a^^,^^b^	4.02±0.39^a^^,^^b^

Note: Compared with the normal and sham-operated groups.

a *p* < 0.001; compared with model group.

b *p* < 0.001.

### Angles of the inclined plate

There was no statistically significant difference in the angle of the inclined plate at different time points compared with the normal and sham-operated groups (*p* > 0.05); The angle of the inclined plate at different time points after injury was lower in the model and training groups than in the normal and sham-operated groups (*p* < 0.05); The angle of the inclined plate at the 14th, 21st, and 28th d after injury was higher in the training group than in the model group (*p* < 0.05) (Table 3).

**Table 3:** Comparison of angles of the inclined plate at different time points in the four groups (¯χ±*S*, °).

Group	Pre-injury	Post-injury				
		1st d	7th d	14th d	21st d	28th d
Normal group	42.52±2.55	41.63±2.41	42.53±3.02	43.16±3.42	42.74±4.02	42.63±3.96
Sham-operated group	42.48±2.34	42.02±3.32	42.41±2.98	42.54±3.41	43.11±4.16	41.59±4.12
Model group	41.89±2.48	15.63±2.24^a^	20.32±3.32^a^	26.32±4.52^a^	30.26±5.52^a^	34.86±3.38^a^
Training group	42.03±2.51	16.03±3.32^a^	21.36±3.54^a^	30.68±5.52^a^^,^^b^	35.69±6.32^a^^,^^b^	39.63±5.12^a^^,^^b^

Note: Compared with the normal and sham-operated groups.

a *p* < 0.001; compared with model group.

b *p* < 0.05.

### Spinal cord and gastrocnemius morphology

**Fig. 2:**
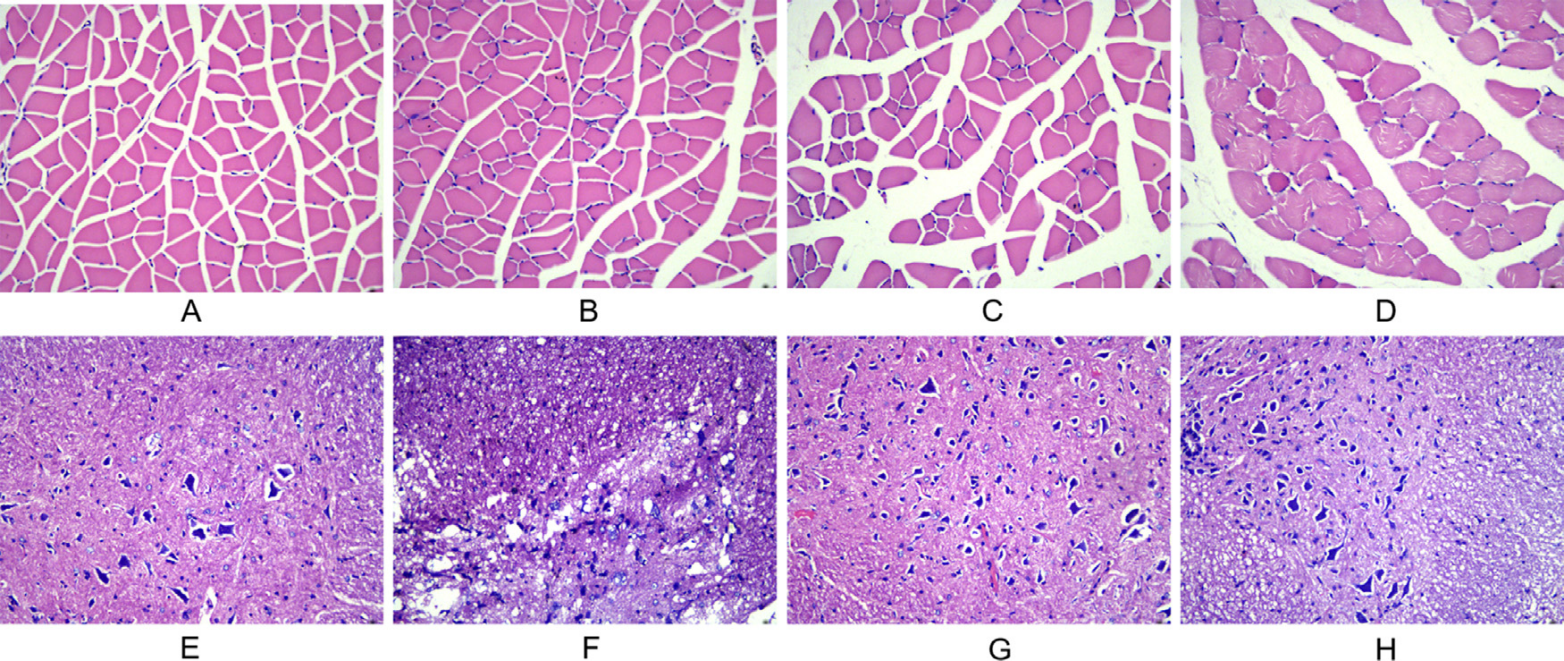
Observation of morphological changes of gastrocnemius muscle and spinal cord histology after spinal cord injury in rats using, HE staining. (A‒D) The morphological changes of gastrocnemius muscle in each group at 28 d after injury (A: normal group; B: sham-operated group; C: model group; D: training group). (E‒H) The morphological changes of spinal cord tissue in each group at 28 d after injury (E: normal group; F: sham-operated group; G: model group; H: training group).

In the normal and sham-operated groups, the muscle fibers were multinucleated giant cells with obtusely angled polyhedral cross-sections, regular morphology of neuronal cells and glial cells, little nuclear chromatin, and light red cytoplasm. In the model group, typical injury changes were observed, showing neuronal deformation and edema, increased inflammatory cells, and thin strips of atrophied muscle fibers, showing cross-sections with sharp angles. In the training group, the injury symptoms were significantly reduced, the muscle fiber atrophy was not obvious, and the structure was basically normal (Fig. 2).

### Cross-sectional area and diameter of muscle fibers

Compared with the normal group and sham-operated group, the differences in muscle fiber cross-sectional area and diameter were not statistically significant (*p* > 0.05); The muscle fiber cross-sectional area and diameter in the model group and training group were lower than those in the normal group and sham-operated group (*p* < 0.05); The muscle fiber cross-sectional area and diameter in the training group were higher than those in the model group (*p* < 0.05) (Table 4).

**Table 4:** Comparison of cross-sectional area and diameter of muscle fibers (¯χ±*S*).

Group	Myofiber cross-sectional area (μm^2^)	Myofiber diameter (μm)
Normal group	55.46±10.19	8.99±1.11
Sham-operated group	54.63±9.65	8.86±0.85
Model group	35.62±8.45^b^	6.03±1.15^b^
Training group	42.53±7.11^a^^,^^c^	7.53±1.43^a^^,^^d^

Note: Compared with the normal and sham-operated groups.

a *p* < 0.01.

b *p* < 0.001; compared with model group.

c *p* < 0.05.

d *p* < 0.01.

### Semi-quantitative values of positive expression of neurospecific markers in spinal cord tissue

MAP1B, NSE, and VIM positive expression semi-quantitative values showed no significant differences between the normal and sham-operated groups (*p* > 0.05); MAP1B, NSE and VIM positive expression semi-quantitative values in the model and training groups were lower than those in the normal and sham-operated groups (*p* < 0.05); MAP1B, NSE and VIM positive expression semi-quantitative values in the training group were higher than those in the model group (*p* < 0.05) (Table 5).

**Table 5:** Comparison of semi-quantitative values of positive expression of neurospecific markers in spinal cord tissues (¯χ±*S*).

Group	MAP1B	NSE	VIM
Normal group	5.63±0.74	5.56±0.82	5.15±0.73
Sham-operated group	5.58±0.72	5.39±0.75	5.21±0.69
Model group	3.38±0.57^a^	3.46±0.62^a^	3.29±0.54^a^
Training group	4.78±0.63^a^^,^^b^	4.53±0.52^a^^,^^b^	4.56±0.63^a^^,^^b^

Note: Compared with normal group and sham-operated group.

a *p* < 0.001; compared with model group.

b *p* < 0.001.

### Expression of TGF-β1, HIF-1α in spinal cord tissue

**Fig. 3:**
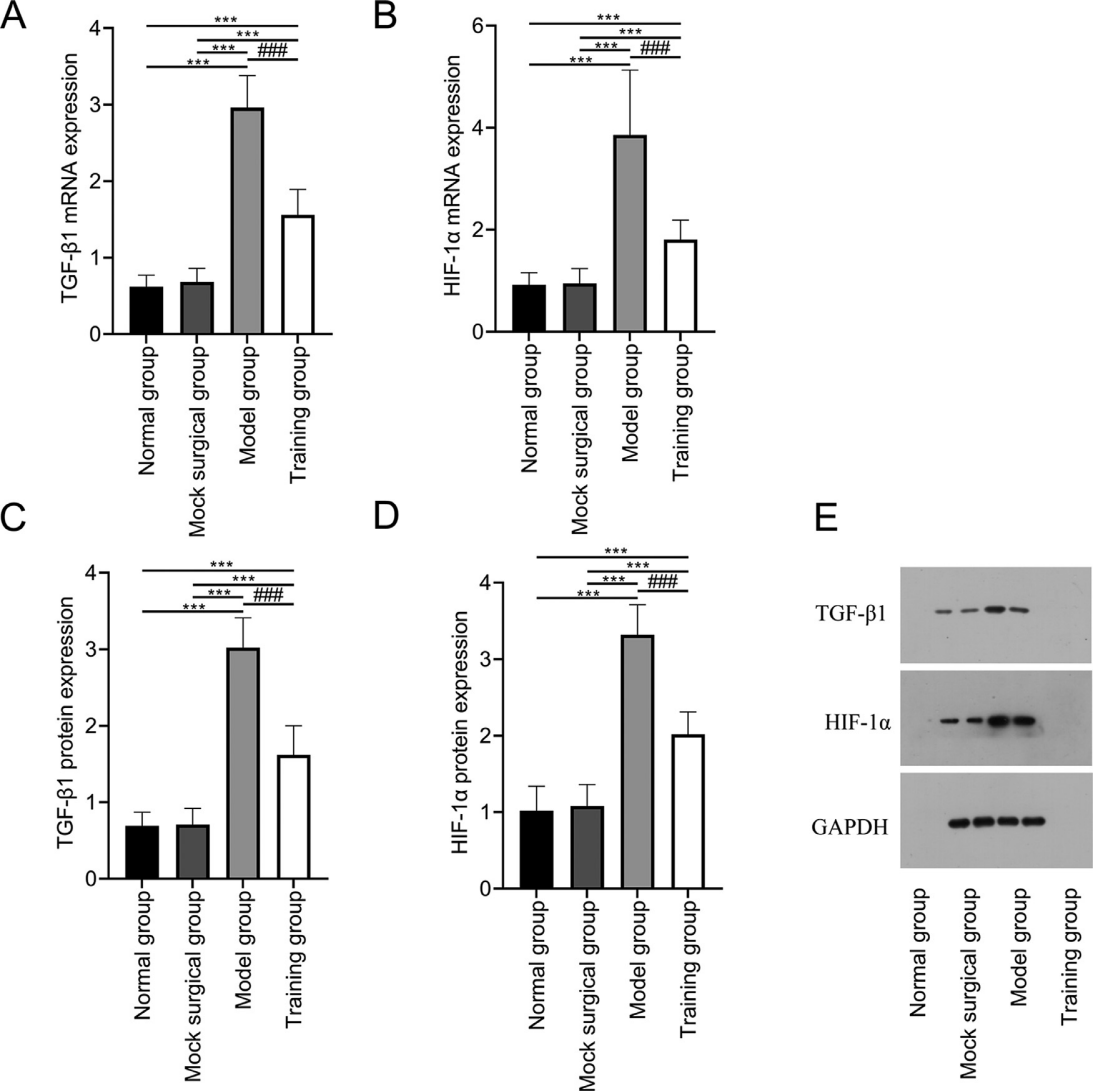
Expression of TGF-β1 and HIF-1α in spinal cord tissue after spinal cord injury in rats. Fig. 3 shows that exercise training significantly reduced the expression of mRNA (A‒B) and protein (C‒D) of TGF-β1 and HIF-1α in spinal cord tissue after spinal cord injury. (E) shows the Western blot band images of TGF-β1 and HIF-1α protein expression. Note: Compared with the normal and sham-operated groups, ^⁎⁎⁎^*p* < 0.001; compared with model group, ^###^*p* < 0.001.

The mRNA and protein expression of TGF-β1 and HIF-1α showed no significant differences between the normal and sham-operated groups (*p* > 0.05); The mRNA and protein expression of TGF-β1 and HIF-1α in the model and training groups were higher than those in the normal and sham-operated groups (*p* < 0.05); The mRNA and protein expression of TGF-β1 and HIF-1α in training group were all lower than those in model group (*p* < 0.05) (Fig. 3).

### Expression of nogo-ngr pathway in spinal cord tissue

**Fig. 4:**
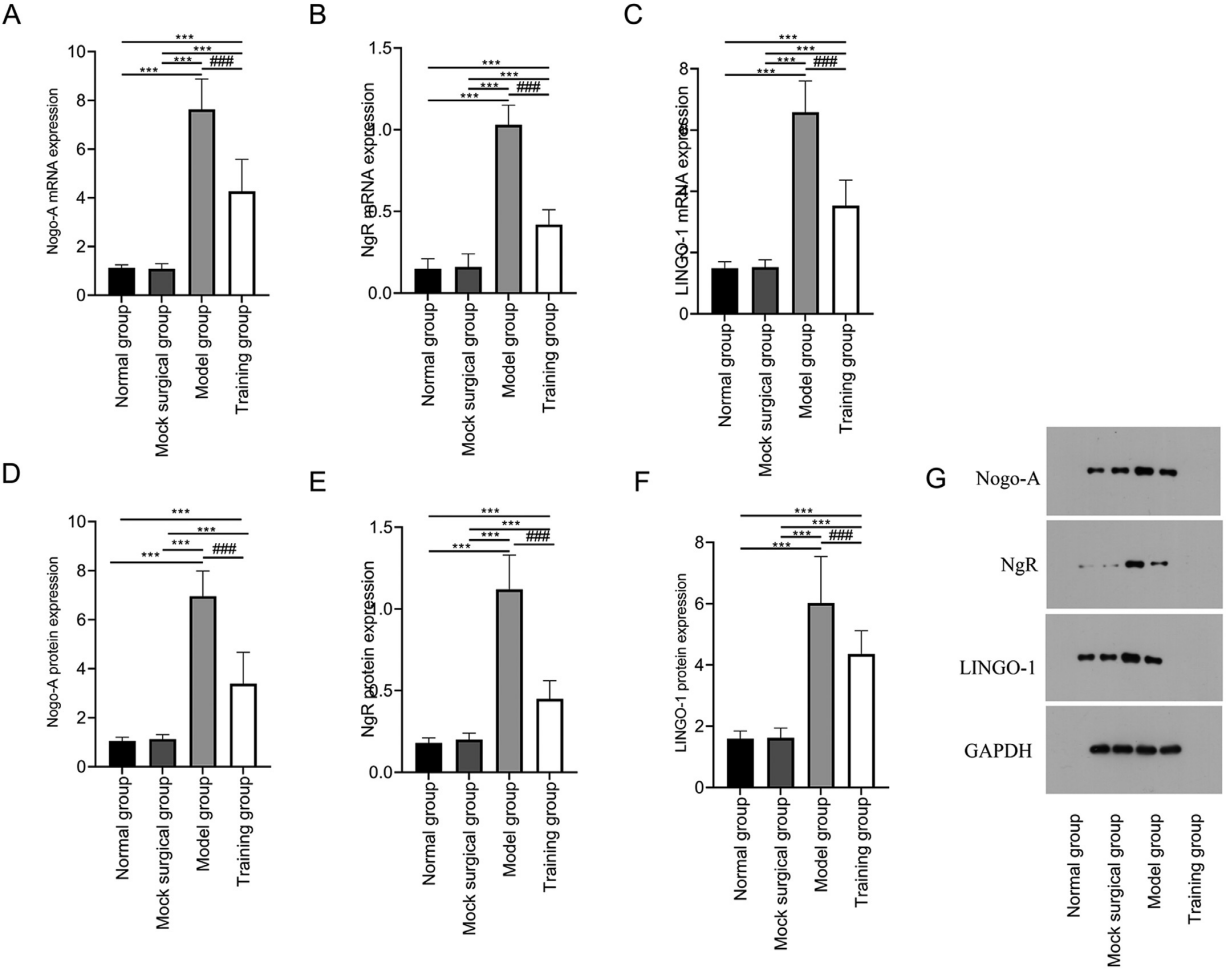
Expression of Nogo-NgR pathway in spinal cord tissue after spinal cord injury in rats. Fig. 4 shows that exercise training significantly reduced the expression of Nogo-A, NgR and LINGO-1 mRNA (A‒C) and protein (D‒F) in spinal cord tissue after spinal cord injury. G shows the Western blot band images of Nogo-NgR pathway protein expression. Note: Compared with the normal and sham-operated groups, ^⁎⁎⁎^*p* < 0.001; compared with model group, ^###^*p* < 0.001.

The mRNA and protein expression of Nogo-A, NgR, and LINGO-1 showed no significant difference between the normal and sham-operated groups (*p* > 0.05); The mRNA and protein expression of Nogo-A, NgR, and LINGO-1 in the model and training groups were higher than those in the normal and sham-operated groups (*p* < 0.05); The mRNA and protein expression of Nogo-A, NgR, and LINGO-1 in the training group were lower in training group than those in the model group (*p* < 0.05) (Fig. 4).

## Discussion

Body weight-supported treadmill training is one of the common exercise training in animal experiments and has been shown to improve the local microenvironment of injury and enhance muscle contraction after neurons are stimulated.^11^,^12^ By using Allen's approach, a rat spinal cord injury model was created for this experiment. The model group had typical spinal cord injuries changes such as neuronal deformation, edema, and increased inflammatory cells, and the cross-section of atrophic muscle fibers was sharp and thin strips. The results were consistent with earlier studies.^13^ The BBB score and modified Tarlow score on the first day after injury were both 0 points.^14^ The BBB score modified Tarlow score, and angle of the inclined plate were all increased in the training group on the 14th, 21st, and 28th d after injury after receiving motor training on the 8th d after injury, which showed that motor training could improve the motor function of rats with spinal cord injury, presumably because motor training could enhance the input of proprioceptive, limb terminal skin sensation and normal motor patterns, activate the central spinal Cord Pattern Generator (CPG), increase the feedback secretion of neurotrophic factors from nerves and muscles, and nourish distal motor neurons, thus promoting the recovery of motor function.^15^ The cross-sectional area and diameter of muscle fibers in the training group were higher than those in the model group, the symptoms of spinal cord injury were significantly reduced, and muscle fiber atrophy was not obvious, and this was consistent with motor improvement, indicating the synchronization of neuromuscular function and motor function recovery after spinal cord injury in rats.

To further analyze the effect of exercise training on neurological function and mechanism of action after spinal cord injury in rats, this study measured the expression of relevant proteins in spinal cord tissues by RT-qPCR and Western Blot method, respectively, and found that the mRNA and protein expression of muscle TGF-β1 and HIF-1α in the training group were lower than those in the model group, and immunofluorescence staining showed that MAP1B, NSE and VIM, specific markers of nerve cells in the training group, was increased, indicating that exercise training could protect the nerve function and promote the healing of vertebral fracture in rats with spinal cord injury. The underlying reasons may be: 1) Exercise training can promote axon collateral bud production and induce functional remodeling of the corticospinal tract; 2) Exercise training can inhibit the release of central inhibitory transmitters such as ɤaminobutyric acid (GABA) and glycine and promote the expression and release of Brain-Derived Nerve Growth Factor (BDNF) and Neurotrophic Factor-3 (NT-3) in the spinal cord, thereby increasing spinal cord plasticity. This improves spinal cord plasticity and promotes neuronal circuit reorganization^3^; Exercise training can improve neurological function by decreasing the inflammatory response and apoptosis in the injured spinal cord, reducing edema, and decreasing the inhibitory effects of related signaling pathways on the proliferation and differentiation of neural stem cells.^16^,^17^

It was discovered that myelin destruction, glial cell loss, and inhibitory factors in glial scars are all linked to the extension of CNS axons. Among these inhibitory factors, myelin-related axon growth inhibitory proteins play a more significant inhibitory role, with Nogo protein acting most prominently.^18^ Nogo-A is Nogo gene's full-length expression and possesses the structural-functional domains Nogo-A-20 and Nogo-A-66, both of which can stabilize the outer fiber skeleton of neurons and prevent cell adhesion and migration by activating the Rho/ROCK pathway and raising Ca2+ levels.^19^ This further causes growth cone collapse and inhibits CNS axons.^20^ NgR is a receptor for Nogo protein inhibition, and the family includes NgR1, NgR2, and NgR3, among which NgR1 needs to bind to the low-affinity neurotrophic factor receptor p75 or TROY or the nervous system-specific transmembrane protein LINGO-1 to co-mediate the myelin inhibitory response because it cannot mediate transmembrane or intracellular signaling.^21^,^22^ After a successful SCI model was established, Nogo-A, NgR, LINGO-1, RhoA, and ROCK II protein and mRNA expression increased in rat spinal cord tissue. Downregulation of Nogo/NgR and Rho/ROCK signaling pathway expression successfully attenuated the inhibition of axonal growth, according to Xiao et al.^23^ Wang^24^ et al. reported that chronic compression of the spinal cord can lead to the loss of anterior horn motor neurons, while inhibition of the Nogo/NgR signaling pathway can promote astrocyte proliferation and synaptic remodeling. This suggests that inhibiting the expression of genes related to the Nogo/NgR pathway may be the key to restoring neural function after spinal cord injury. The results of this study found that the mRNA and protein expression of Nogo-A, NgR, and LINGO-1 in the model and training groups were higher than those in the normal and sham-operated groups, and it can be found that spinal cord injury is the direct cause of activation of the Nogo/NgR signaling pathway, and the reason may be related to a series of biochemical cascade reactions at the molecular and cellular levels initiated by primary injury in the early stages of spinal cord injury, causing impaired microcirculation, tissue ischemia, axonal demyelination, lipid peroxidation response, and apoptosis^25^; and Nogo-A binding to the corresponding receptor NgR activates the downstream Rho/ROCK pathway, altering the cone local actin cytoskeleton and growth cone morphology and stability, which inhibits axonal regeneration.^26^ The mRNA and protein expression of Nogo-A, NgR, and LINGO-1 in the training group were lower than those in the model group, indicating that exercise training can inhibit the Nogo-NgR signaling pathway in rats with spinal cord injury and induce a microenvironment suitable for neuronal axon repair, thereby promoting axon repair and neurite growth.

In conclusion, exercise training can enhance motor function and improve spinal cord and gastrocnemius muscle morphology, promote neurological recovery after spinal cord injury in rats, promote motor function recovery of the hind limb, regulate the expression of neurospecific markers, TGF-β1 and HIF-1α in spinal cord tissues, and its mechanism of action may be related to the inhibition of Nogo-NgR signaling pathway to promote neuronal growth.

## Funding

This research did not receive any specific grant from funding agencies in the public, commercial, or not-for-profit sectors.

## CRediT authorship contribution statement

**Xubin Ji:** Conceptualization, Data curation, Formal analysis, Methodology, Investigation, Writing – original draft. **Zhaowan Xu:** Methodology, Formal analysis, Validation. **Dayong Liu:** Methodology, Formal analysis, Validation. **Yangwang Chen:** Conceptualization, Investigation, Formal analysis, Methodology, Writing – review & editing.

## Conflicts of interest

The authors declare no conflicts of interest.

## References

[1] Anjum A., Yazid M.D., Fauzi Daud M., Idris J., Ng A.M.H., Selvi Naicker A. (2020). Spinal Cord Injury: pathophysiology, multimolecular interactions, and underlying recovery mechanisms. Int J Mol Sci.

[2] Fouad K., Popovich P.G., Kopp M.A., Schwab J.M. (2021). The neuroanatomical-functional paradox in spinal cord injury. Nat Rev Neurol.

[3] Marquez-Chin C., Popovic M.R. (2020). Functional electrical stimulation therapy for restoration of motor function after spinal cord injury and stroke: a review. Biomed Eng Online.

[4] Yıldırım M.A., Öneş K., Gökşenoğlu G. (2019). Early term effects of robotic assisted gait training on ambulation and functional capacity in patients with spinal cord injury. Turk J Med Sci.

[5] Jo H.J., Perez M.A. (2020). Corticospinal-motor neuronal plasticity promotes exercise-mediated recovery in humans with spinal cord injury. Brain.

[6] Unger J., Chan K., Scovil C.Y., Craven B.C., Mansfield A., Masani K. (2019). Intensive Balance Training for Adults With Incomplete Spinal Cord Injuries: protocol for an Assessor-Blinded Randomized Clinical Trial. Phys Ther.

[7] Ni S., Yang B., Xia L., Zhang H. (2021). EZH2 Mediates miR-146a-5p/HIF-1α to Alleviate Inflammation and Glycolysis after Acute Spinal Cord Injury. Mediators Inflamm.

[8] Lu X.M., Mao M., Xiao L., Yu Y., He M., Zhao G.Y. (2019). Nucleic Acid Vaccine Targeting Nogo-66 Receptor and Paired Immunoglobulin-Like Receptor B as an Immunotherapy strategy for spinal cord injury in rats. Neurotherapeutics.

[9] Kilkenny C., Browne W., Cuthill I.C., Emerson M., Altman D.G. (2010). NC3Rs Reporting Guidelines Working Group. Animal research: reporting in vivo experiments: the ARRIVE guidelines. Br J Pharmacol.

[10] Wu Y.J., Hou Y.N., Zhang Z.T., Liu Z.P., Nie Z.H., Fan G.L. (2016). Early exercise training combined with neural stem cell transplantation improves hindlimb motor function after spinal cord injury in rats. Chin J Tissue Eng Res.

[11] Ilha J., Meireles A., de Freitas G.R., do Espírito Santo C.C., Machado-Pereira N., Swarowsky A. (2019). Overground gait training promotes functional recovery and cortical neuroplasticity in an incomplete spinal cord injury model. Life Sci.

[12] Vivodtzev I., Taylor J.A. (2021). Cardiac, autonomic, and cardiometabolic impact of exercise training in spinal cord injury: a qualitative review. J Cardiopulm Rehabil Prev.

[13] Wu J., Gao J.X., Liu K.J., Zhang R.H. (2006). Spinal cord injury on motor capacity and its pathology in rats. J Shandong Univ.

[14] de Freitas G.R., Szpoganicz C., Ilha J. (2018). Does Neuromuscular Electrical Stimulation Therapy increase voluntary muscle strength after spinal cord injury? A systematic review. Top Spinal Cord Inj Rehabil.

[15] Kryger M.A., Crytzer T.M., Fairman A., Quinby E.J., Karavolis M., Pramana G. (2019). The effect of the interactive mobile health and rehabilitation system on health and psychosocial outcomes in spinal cord injury: randomized controlled trial. J Med Internet Res.

[16] Hubscher C.H., Wyles J., Gallahar A., Johnson K., Willhite A., Harkema S.J. (2021). Effect of different forms of activity-based recovery training on bladder, bowel, and sexual function after spinal cord injury. Arch Phys Med Rehabil.

[17] Zaaya M., Pulverenti T.S., Knikou M. (2021). Transspinal stimulation and step training alter function of spinal networks in complete spinal cord injury. Spinal Cord Ser Cases.

[18] Hui S.P., Monaghan J.R., Voss S.R., Ghosh S. (2013). Expression pattern of Nogo-A, MAG, and NgR in regenerating urodele spinal cord. Dev Dyn.

[19] Liu H.C. (2021). The new functional domain of Nogo-A promotes inflammatory pain and inhibits neurite growth by binding to NgR1. Chin J Pain Med.

[20] Jiang J., Yu Y., Zhang Z., Ji Y., Guo H., Wang X. (2021). Effects of Nogo-A and its receptor on the repair of sciatic nerve injury in rats. Braz J Med Biol Res.

[21] Harel N.Y., Song K.H., Tang X., Strittmatter S.M. (2010). Nogo receptor deletion and multimodal exercise improve distinct aspects of recovery in cervical spinal cord injury. J Neurotrauma.

[22] Hu H., Wang H., Liu W. (2021). Effect of ganglioside combined with Chip Jiaji electro-acupuncture on Nogo-NgR signal pathway in SCI rats. Saudi J Biol Sci.

[23] Xiao W.P., Ding L.L., Min Y.J., Yang H.Y., Yao H.H., Sun J. (2019). Electroacupuncture promoting Axonal Regeneration in spinal cord injury rats via suppression of Nogo/NgR and Rho/ROCK signaling pathway. Neuropsychiatr Dis Treat.

[24] Wang Y., Sun J.C., Wang H.B., Xu X.M., Yang Y., Kong Q.J. (2018). Effects of MicroRNA-494 on Astrocyte Proliferation and Synaptic Remodeling in the spinal cord of a rat model of chronic compressive spinal cord injury by regulating the Nogo/Ngr signaling pathway. Cell Physiol Biochem.

[25] Li X., Wang Q., Ding J., Wang S., Dong C., Wu Q. (2020). Exercise training modulates glutamic acid decarboxylase-65/67 expression through TrkB signaling to ameliorate neuropathic pain in rats with spinal cord injury. Mol Pain.

[26] Wang P., Yin R., Wang S., Zhou T., Zhang Y., Xiao M. (2021). Effects of repetitive transcranial magnetic stimulation (rTMS) and treadmill training on recovery of motor function in a rat model of partial spinal cord injury. Med Sci Monit.

